# Lung tissue biomechanics imaged with synchrotron phase contrast microtomography in live rats

**DOI:** 10.1038/s41598-022-09052-9

**Published:** 2022-03-23

**Authors:** Jose-Luis Cercos-Pita, Luca Fardin, Hugo Leclerc, Bertrand Maury, Gaetano Perchiazzi, Alberto Bravin, Sam Bayat

**Affiliations:** 1 Hedenstierna Laboratory, Department of Surgical Sciences, Uppsala University, Uppsala, Sweden; 2 European Synchrotron Radiation Facility, Grenoble, France; 3 Laboratoire de Mathématiques d’Orsay, Université Paris-Saclay, Orsay, France; 4 Département de Mathématiques Appliquées, Ecole Normale Supérieure, Université PSL, Paris, France; 5 Physics Department, Milano Bicocca University, Milan, Italy; 6 Synchrotron Radiation for Biomedicine STROBE Inserm UA07, Univ. Grenoble Alpes, Grenoble, France; 7 Univ. Grenoble Alpes - Inserm UA07, Synchrotron Radiation for Biomedicine (STROBE) Laboratory, 2280 Rue de la Piscine, 38400 Grenoble, France

**Keywords:** Physiology, Respiration, Respiratory distress syndrome, Engineering, Biomedical engineering

## Abstract

The magnitude and distribution of strain imposed on the peripheral airspaces by mechanical ventilation at the microscopic level and the consequent deformations are unknown despite their importance for understanding the mechanisms occurring at the onset of ventilator-induced lung injury. Here a 4-Dimensional (3D + time) image acquisition and processing technique is developed to assess pulmonary acinar biomechanics at microscopic resolution. Synchrotron radiation phase contrast CT with an isotropic voxel size of 6 µm^3^ is applied in live anesthetized rats under controlled mechanical ventilation. Video animations of regional acinar and vascular strain are acquired in vivo. Maps of strain distribution due to positive-pressure breaths and cardiovascular activity in lung acini and blood vessels are derived based on CT images. Regional strain within the lung peripheral airspaces takes average values of 0.09 ± 0.02. Fitting the expression *S* = *kV*^*n*^, to the changes in peripheral airspace area (*S*) and volume (*V*) during a positive pressure breath yields an exponent n = 0.82 ± 0.03, suggesting predominant alveolar expansion rather than ductal expansion or alveolar recruitment. We conclude that this methodology can be used to assess acinar conformational changes during positive pressure breaths in intact peripheral lung airspaces.

## Introduction

Mechanical ventilation used during anaesthesia or in acute respiratory failure causes or worsens strain-induced lung tissue injury^1^. It is generally agreed that excessive mechanical strain due to inflation^2^, cyclic recruitment-derecruitment^3^, increased energy dissipation within the parenchyma^4^ and associated inflammation^5^ are contributing factors. The magnitude and distribution of strain imposed on the peripheral airspaces by mechanical ventilation at the microscopic level and the resulting deformation of peripheral airspaces are unknown despite their importance for understanding the mechanisms occurring at the onset of ventilator-induced lung injury (VILI). Indeed, a fundamental question that has remained elusive for decades is how the lung acinus expands with lung inflation^6^. This question is particularly pressing in the context of severe acute respiratory syndrome due to coronavirus-2 pandemic (SARS-CoV-2) where VILI has been recognized as a contributing factor to patient morbidity and mortality^7^.

Imaging methods for high resolution in vivo assessment of peripheral airspace deformation dynamics are lacking. The reason lies in the difficulty of determining effective structural deformation due to the motion blurring at microscopic resolution, which is induced by respiration and cardiovascular activity. Different imaging modalities such as optical coherence tomography (OCT)^8,9^, ultrasound (US)^10^, magnetic resonance tomography^11^ (MRI) and X-ray computed tomography (CT)^12^ have been used to quantify regional tissue strain, or the normalized deformation of a tissue that changes shape or volume following mechanical loading over time. Because of the limitations in tissue penetration (US, OCT) and spatial resolution (US, MRI), X-ray imaging is the most suitable modality for imaging the lung tissue morphology. However, in its practical implementation, a compromise is made between the spatial resolution, temporal resolution, size of the imaging field and sensitivity.

To minimize motion blurring occurring in vivo, cardiac gating methods have been employed where the electrocardiographic activity is monitored and image acquisition is synchronized with a given phase of the cardiac cycle. Using a prospective cardiac gating technique, Lovric et al. were able to image mouse lungs at 1.1 µm voxel size during static breath hold conditions^13^. By changing the level of positive pressure at the airway opening, they were able to resolve alveolar structures at different levels of inflation. However, imaging the lung in static conditions is less physiological and does not allow capturing the full scope of local lung mechanics. This is because the lung tissue is viscoelastic, meaning that its apparent elastic properties depend on the rate of volume change^14^.

To circumvent this limitation, dynamic four-dimensional (3D + time) x-ray CT (4D-CT) imaging techniques have been developed^15–18^. This approach refers to sequential acquisition of 3D images with prospective gating or retrospective sorting of image projections with respect to a periodic motion. Available methods based on X-ray attenuation using commercial x-ray sources have significant limitations arising from low available X-ray flux, which reduces spatial resolution for a given signal-to-noise ratio, and prolongs acquisition time. Clinical 4DCT combined with image registration of images at 16 time points within the respiratory cycle has been used to assess regional lung expansion and strain in humans, at voxel dimensions of ~ 0.5 mm^19^, which does not allow resolving alveolar structures. Besides motion blurring, another limitation of high-resolution CT imaging is that the lung being an air-filled organ, it weakly attenuates x-rays which reduces sensitivity. Previously, dynamic phase-contrast 4DCT utilizing x-rays produced with a synchrotron source have been used to asses lung tissue motion and regional expansion in in vivo rabbit pups^20^ and mice^21^. This technique takes advantage of the high intensity and coherence of synchrotron radiation to acquire 3D images of the lung at resolutions of 15 µm voxel size and 16 time points per respiratory cycle^20^ and up to 50 time points per respiratory cycle at 20 µm voxel size^21^.

One approach to describe how the conformation of terminal airspaces is altered by inflation, is to determine the changes in the surface area of airspaces in proportion to volume, by fitting the expression *S* = *kV*^*n*^, to the changes in peripheral airspace area (*S*) and volume (*V*) during a positive pressure breath, where *k* is a constant, and *n* the exponent of volume changes with respect to the change in surface area. With a perfectly isotropic expansion, surface area necessarily increases in proportion to V^2/3^ (n = 0.67) because surface area varies as the square and volume as the cube of a characteristic linear dimension. Departure from this value would indicate a configurational change of the acinus with inflation^22^. Assuming an acinar structure with an axial duct from which alveoli open radially, the relative shape and changes in ductal and alveolar volumes result in different overall surface changes relative to volume. Based on this approach in lungs fixed at different volumes, Gil et al.^23^ described different mechanisms of acinar configurational changes (S ∝ V^n^): (1) sequential recruitment-derecruitment (n = 1); (2) isotropic balloonlike inflation (n = 0.67); (3) simultaneous change in alveolar size and shape, e.g.: from dodecahedral to spherical, due to alveolar septal unpleating (n > 0.67); (4) crumpling of the alveolar surface with anisotropic, accordion-like deformations of the entire peripheral airspace units (n = 1). The results of Gil et al. suggested that although all these mechanisms are involved, they tended to occur at different lung volumes. At lower lung volumes, deformation occurred predominantly within the alveolar ducts (S = kV^0.33^), while at higher lung volumes, alveolar inflation predominated, with simultaneous change in alveolar shape and volume. Because of the limitations of current imaging techniques, to date this issue has not been investigated in deep lung acini in vivo and in dynamic conditions^6^.

Here we extend 4D-CT of lung tissue biomechanics to smaller length scales and higher temporal resolution than previously achieved, in live anesthetized rats under controlled mechanical ventilation. We use phase-contrast imaging with radiation produced with a synchrotron source, and a combination of technological advances including a fast-imaging camera connected to optics allowing an isotropic voxel size of 6 µm^3^ ^24^. We applied this methodology to acquire maps of strain distribution due to positive-pressure breaths in the lung acini and in blood vessels. We show that this methodology can be used to assess conformational changes during positive pressure breaths in intact peripheral lung airspaces in vivo.

## Results

### Dynamic synchrotron phase contrast in vivo microtomography

**Figure 1:**
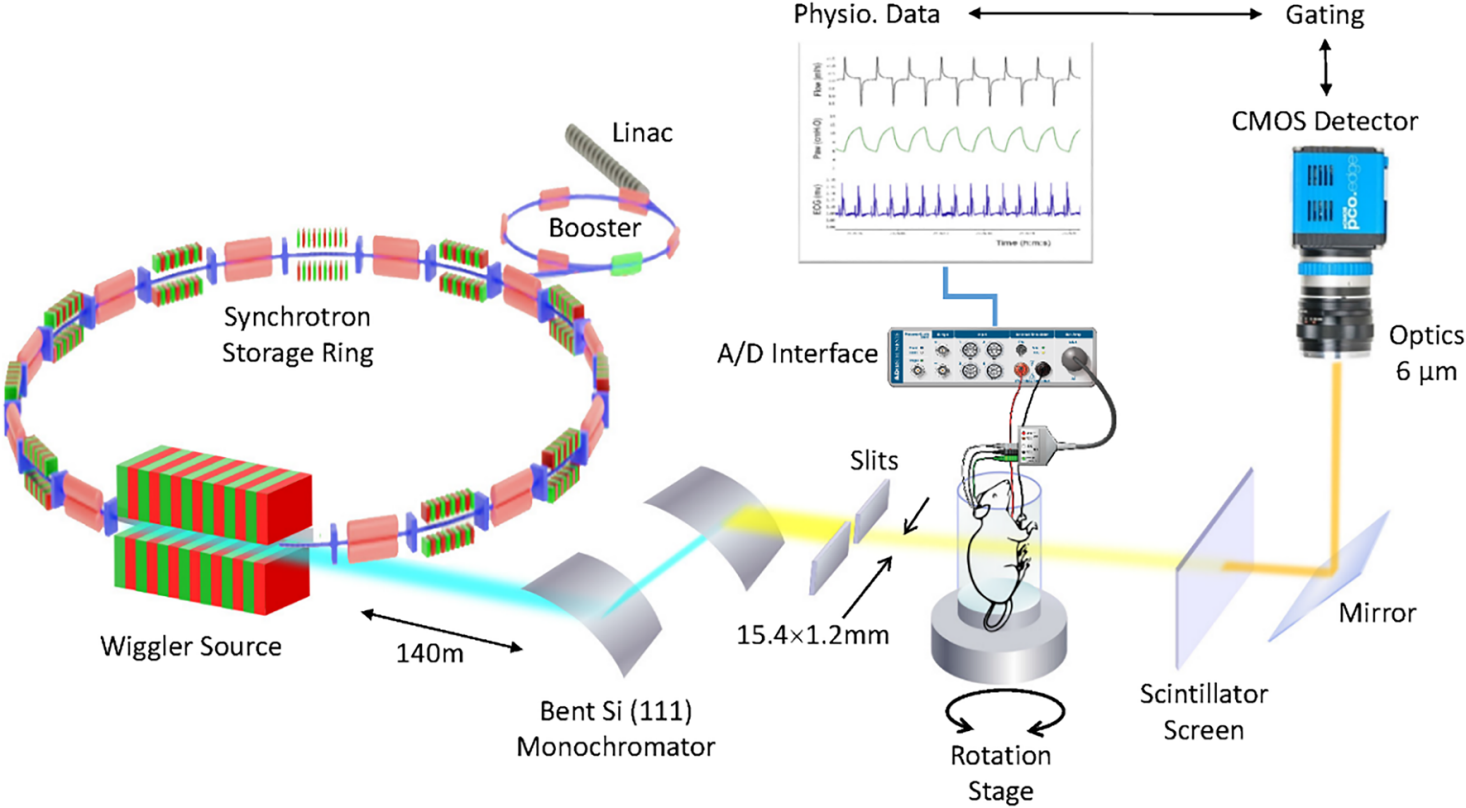
Dynamic 3D microscopy of rat lung using a synchrotron X-ray source. High-intensity coherent X-rays generated from electrons orbiting in a storage ring, are rendered monochromatic using bent silicon crystal optics, and detected by a PCO Edge 5.5 camera coupled to a Cerium-doped Lutetium Aluminium Garnet (LuAG:Ce) scintillator and optics yielding an isotropic pixel size of 6 µm^3^. The anesthetized rat is mechanically ventilated while the electrocardiogram and respiration are monitored and recorded.

In this study, we used phase-contrast imaging with radiation produced with a synchrotron source. Due to its high intensity, which is orders of magnitude higher than conventional sources, synchrotron radiation allows the temporal and spatial resolution of 4D-CT imaging to be improved, performing 4D 1–10 µm^3^ voxel size CT imaging in the temporal scale of minutes. Due to the strong cardiac-induced deformations, the alveolar structures often do not return to the same position at end-expiration, which makes dynamic tomography very challenging at micrometric spatial resolutions, using 4DCT^17^. Here we used a combination of a fast-imaging camera connected to optics allowing an isotropic voxel size of 6 µm^3^ (Fig. 1), combined with triggering of breathing by the electrocardiographic and respiratory signals. We implemented an open software environment for 3D mapping of lung acinar and vascular biomechanics. We acquired 4D volumetric phase-contrast microtomographic images in 3 healthy anesthetized and mechanically ventilated rats in vivo. Sequential 3D CT images covering a field of view of 15.36 × 15.36 × 1.2 mm^3^ were reconstructed at 10 ms time intervals over one entire breath lasting 760 ± 10 ms. This allowed reconstructing 78 successive time points within a single breath. We show a video animation of the structural deformation of the lung within the respiratory cycle (Supporting video 1).

### Mapping dynamic strain within lung airspaces and blood vessels

**Figure 2:**
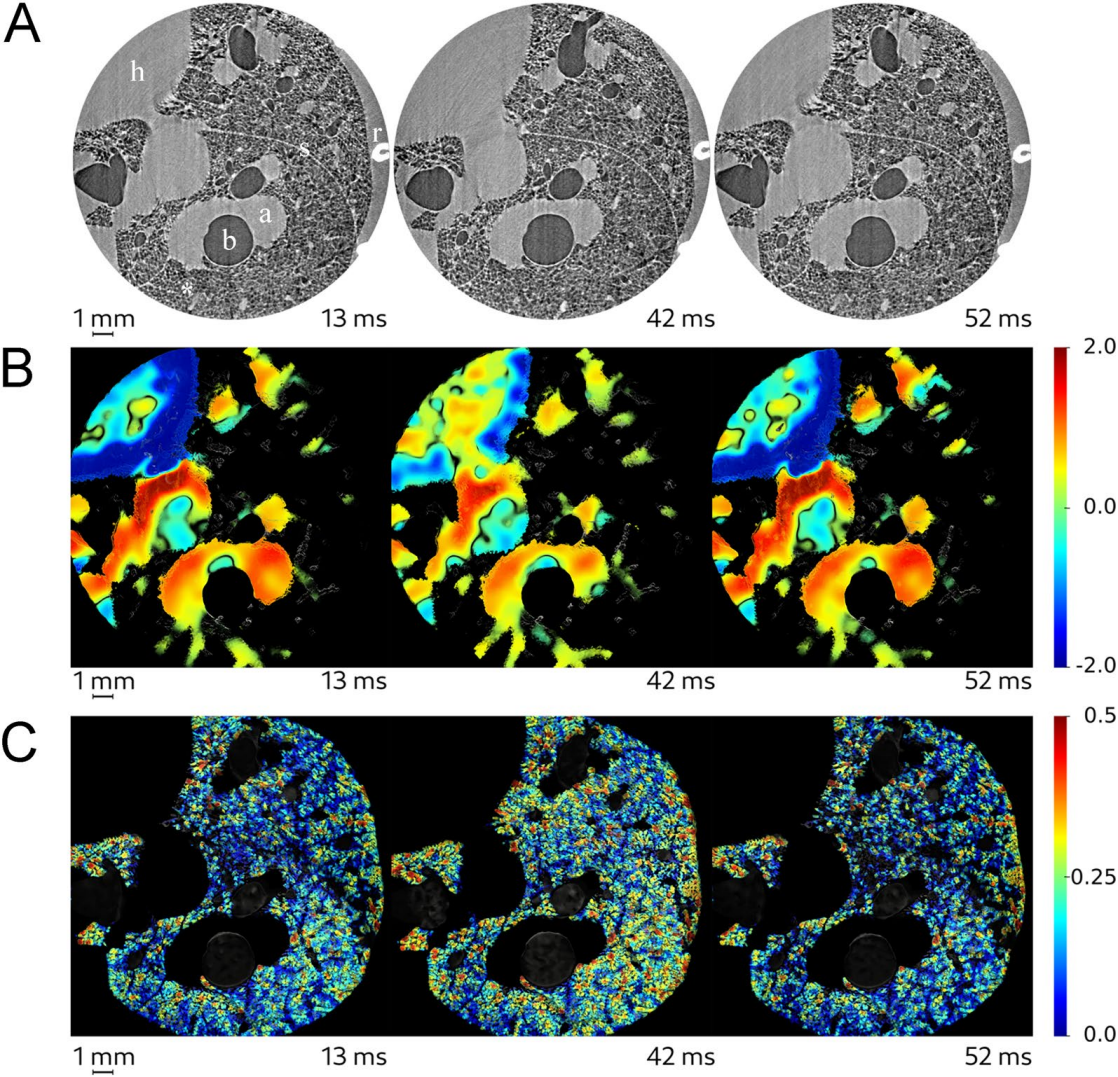
Quantitative mapping of lung tissue biomechanics in a live rat. (**A**) Sample sequential x-ray phase-contrast CT images at successive time points, reconstructed by retrospectively sorting of 250,000 individual image projections with respect to the phase of heart contraction and breathing, *b*: bronchus, *a*: artery, *h*: heart, *s*: interlobar scissure, *r*: rib, *: alveoli; (**B**) sequential local strain maps of vascular structures; (**C**) sequential regional strain maps of airspaces. Color bars indicate strain (*δV/V*_*t0*_, where *t0* is the start of the breath). Corresponding video animations are provided in the online supplement.

**Figure 3:**
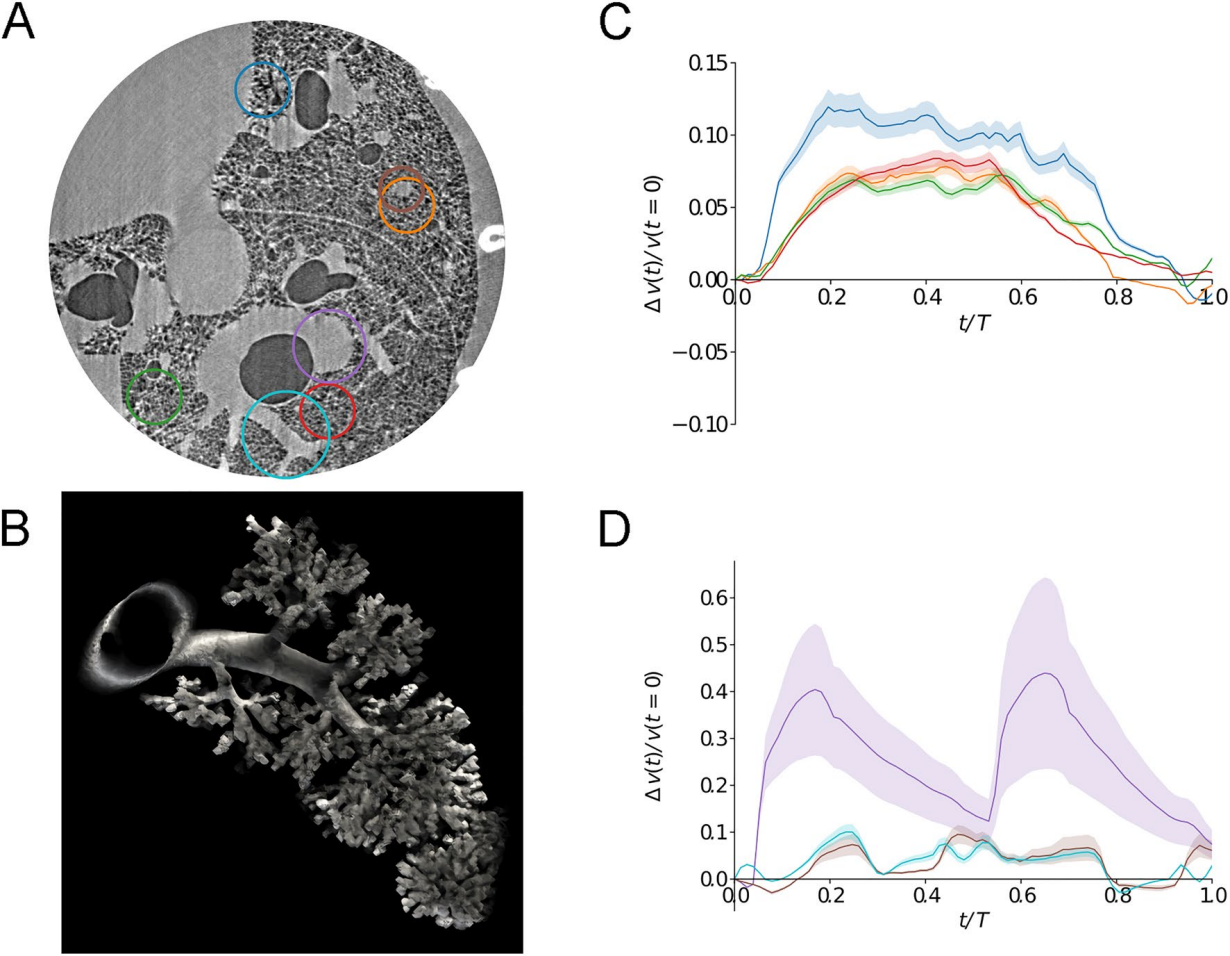
(**A**) X-ray phase-contrast CT image of live rat lung. Colored circles show regions of interest where regional biomechanics are computed; (**B**) a segmented airway with subtending conducting airways and terminal acinar structures at end-expiration in a live rat; (**C**) regional strain as a function of time computed within airspaces in the regions of interest of same color as in (**A**). The shaded area represents within-ROI standard deviation; (**D**) regional strain as a function of time (*t*), expressed relative to the total breath duration (*T*), computed within blood vessels in the regions of interest shown in (**A**). The shaded area represents within-ROI standard deviation. Note the typical pulse-wave deformation within the larger artery which is significantly damped in smaller caliber vessels.

Dynamic strain maps of airspaces and blood vessels are shown in Supporting video 2 and 3, respectively. Mean recorded mechanical ventilation parameters were: maximal airway pressure (Pmax): 11.6 ± 1.1 cmH_2_O; positive end-expiratory pressure (PEEP): 5.9 ± 0.3 cmH_2_O; driving pressure (Pmax – PEEP): 5.7 ± 0.8 cmH_2_O; respiratory rate: 72 ± 7.2 bpm; tidal volume: 2.8 ± 0.2 ml/kg. Figure 2 shows 2D axial slices of the lung morphology, as well as maps of local strain within the airspaces and vascular structures at 3 successive time points within the respiratory cycle. The selected ROIs in one animal are shown in Fig. 3A. Figure 3B demonstrates the quality of a sample acinar segmentation. Figure 3C, D show the evolution of strain within the selected ROIs in airspaces and blood vessels, respectively. We found an average maximal regional lung strain within the airspaces of 0.09 ± 0.02. Within the blood vessels, it is interesting to note that within the larger pulmonary artery, a ‘docrotic notch’ due to to the closure of the pulmonary valves at end-systole is clearly visible, while this waveform is damped within the smaller vascular branches (Fig. 3D).

Previously, Yen et al.^25^ imaged whole mouse lungs in vivo, using a liquid metal jet laboratory X-ray source at 30 frames/s at ~ 50 µm resolution, which allowed assessing the regional tidal lung expansion. Although large-scale regional lung expansion inhomogeneity in between regions about the size of lobes is well known, much less data is available on the deformation of the lung tissue at subacinar scales. In this study we imaged a region that represents ~ 10% of the healthy rat lung volume at end-expiration. The size of the field of view, which was determined by the dimensions of the X-ray beam and the characteristics of the detection system, allowed to fully assess deep lung structures in a representative sample of the lung. Assessing other lung regions would require repeating the scan. Imaging the entire lung dynamically by 4DCT at this spatial and temporal resolution using larger beam dimensions and field of view is limited by the amount of data that is generated, that needs to be transferred, stored and processed, which is on the order of several TB for a single scan. Nevertheless, future studies should allow studying how microscopic strain distribution in the lung airspaces and blood vessels determines structural damage and inflammation, under mechanical ventilation.

### Ventilation-induced changes in lung airspace conformation

**Figure 4:**
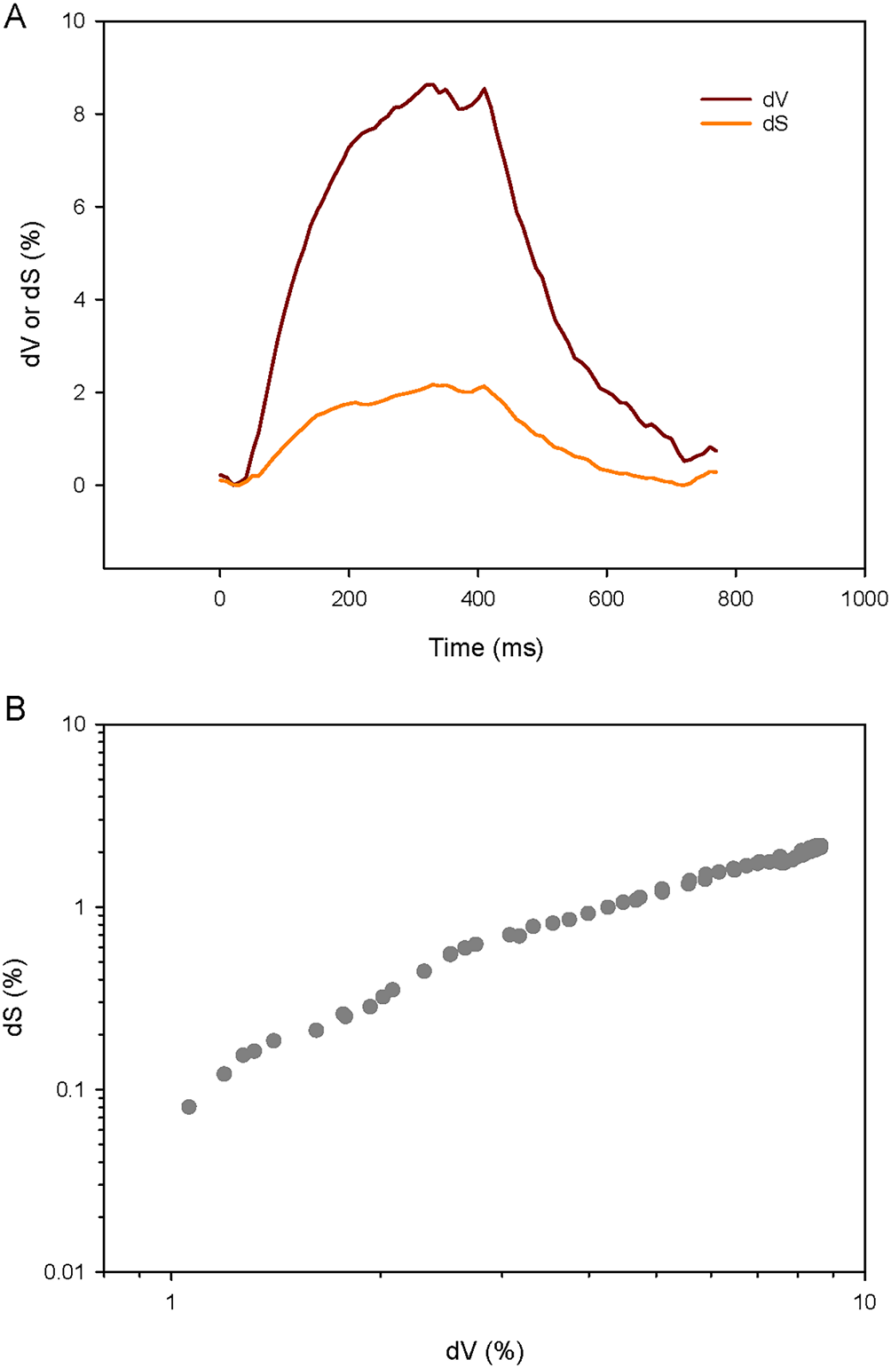
(**A**) Relative surface and volume change of peripheral airspaces within a sample ROI (green circle in Fig. 3A excluding the bronchus); (**B**) Log–Log relation between changes in surface (S) and volume (V). A fit of this relationship by linear regression yields the exponent *n* and a constant *k* in *S* = *kV*^*n*^.

The evolution of the volume (V) and internal surface area (S) of the segmented acini within a representative ROI throughout a respiratory cycle is shown in Fig. 4, as well as a log–log plot of S against V. Table 1 shows the values of the exponent *n* calculated by fitting the expression $S=k{V}^{n}$, to the changes in acinar surface area and volume during a positive pressure breath from an average PEEP of 6 to an average peak airway pressure of 12 cmH_2_O and back. The mean value of this exponent was 0.82 ± 0.03 (n = 12 ROI in 3 animals, Table 1). The 12 ROI assessed here are considered as a representative sample of deep-lung alveoli.

**Table 1:** Values of exponent *n* calculated by fitting the expression $S = kV^{n}$, to the changes in acinar surface area (S) and volume (V) during a positive pressure inspiration from 6 to 12 cmH_2_O.

Rat	ROI^a^	n
R7	1	0.85
R7	2	0.82
R7	3	0.85
R7	4	0.81
R7	5	0.80
R8	1	0.80
R8	2	0.76
R8	3	0.84
R9	1	0.87
R9	2	0.84
R9	3	0.81
R9	4	0.84
m ± SD		0.82 ± 0.03

^a^Region of interest.

The changes in lung acinar shape as a function of lung inflation have been assessed post-mortem in fixed or frozen tissue samples using light microscopy and electron microscopy^26–28^. Post-mortem light microscopic measurements of fixed or frozen lungs have found conflicting results regarding the configurational changes of lung acini with lung inflation. Some have suggested that alveoli expand isotropically^29^. This means that linear dimensions of alveolar structures vary as the cube root of lung volume (V^1/3^). Others however, have found some degree of anisotropic expansion with the alveolar duct volume changing proportionately more than alveolar volume^30^. Previously, Carney et al. investigated the mechanical behavior of subpleural alveoli during lung inflation in vivo, using intravital microscopy through a thoracotomy incision in dogs^8^. They found that above 20% TLC, alveolar volume remained stable while an increasing number of alveoli were recruited and concluded that lung volume changes predominantly occurred as a result of alveolar recruitment. However, in their study alveolar mechanics was analyzed in initially degassed lungs in open chest animals which may have promoted alveolar collapse. Using intravital microscopy and optical coherence microscopy (OCT), Mertens et al.^31^ investigated alveolar dynamics through a pleural window in open-chest mice with normal and injured lungs. They found that alveolar distension rather than recruitment was the main mechanism of lung inflation. Besides species and methodological differences that may explain the discrepancy in the data from intravital microscopy in these and other studies^32–34^, there has been concern that the mechanical behavior of subpleural alveoli may be different from those deep in the lung.

**Figure 5:**
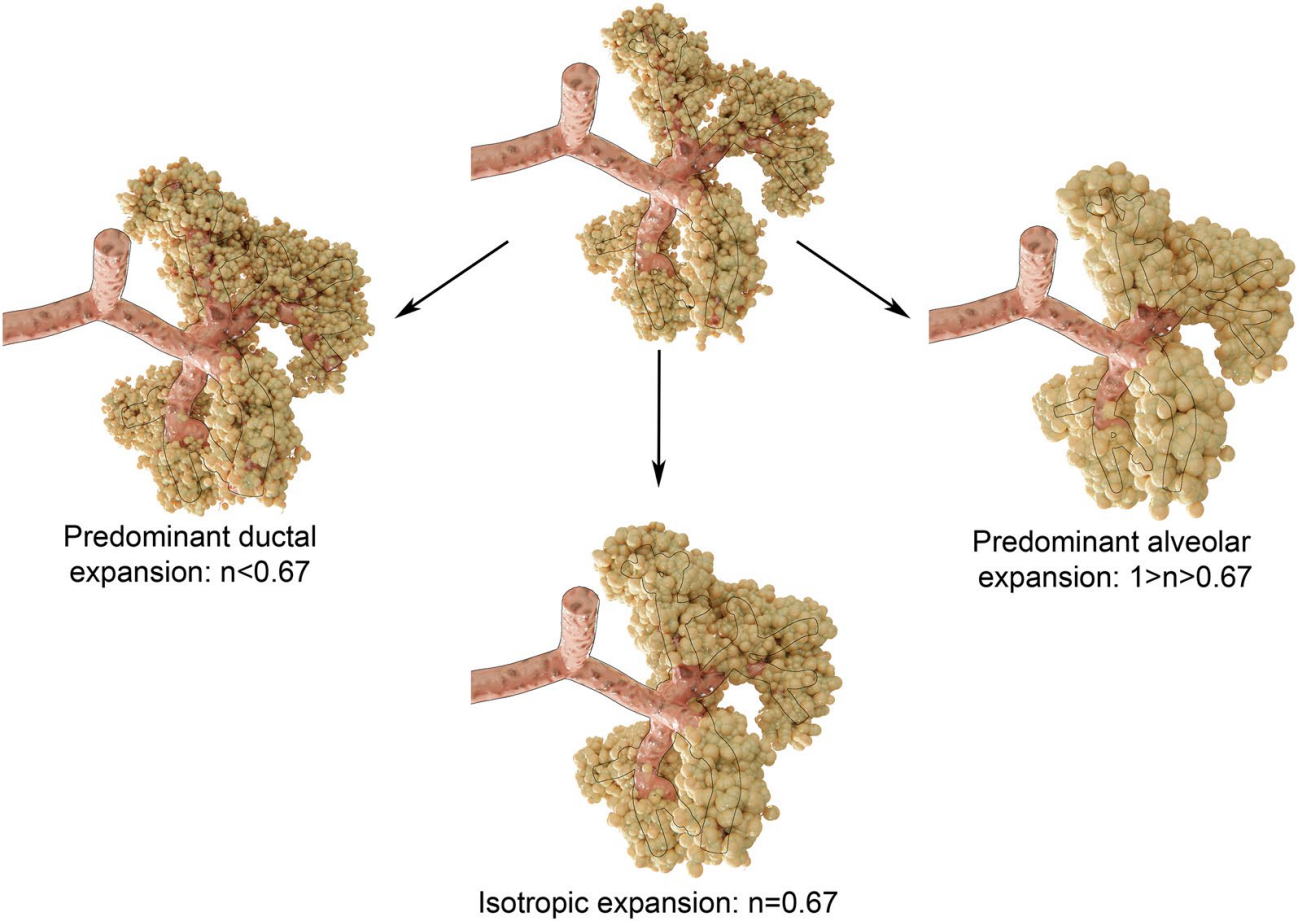
Schematic representation of how different acinar conformational changes translate into the exponent n in *S* = *kV*^*n*^*.* Thin black lines delineate approximate alveolar ductal size. Isotropic balloon like expansion results in *S* = *kV*^*2/3*^, while a predominantly ductal expansion (50% increase in radius) would cause n to be < 2/3 or < 0.67, and a predominantly alveolar expansion (50% increase in radius) would increase n > 0.67.

Sera et al.^35^ investigated lung acinar dynamics in post-mortem mice without tissue fixation in static conditions, using synchrotron phase-contrast microtomography. They found that the alveolar duct diameter changed dramatically during inflation at low pressures and remained relatively constant above an airway pressure of ~ 8 cmH_2_O, where alveolar inflation predominated with S = kV^0.87^ ^35^. Lovric et al.^36^ studied alveolar expansion in post-mortem mouse lungs also using synchrotron phase-contrast microtomography at static pressures of 10–30 cmH_2_O. Their results indicated inflation of the existing alveoli, without evidence of recruitment, however, they did not measure the changes in surface area with respect to volume. The findings of the present study (S = kV^0.82^) while obtained in vivo and in dynamic conditions under realistic mechanical ventilation settings are very similar to those of Sera et al. at airway pressures > 8 cmH_2_O. This finding is indicative of an anisotropic behavior of the overall lung acinus, where the stress–strain relation in alveolar ducts, mainly determined by high densities of connective tissue situated around the alveolar mouths^37–39^, is different from that of alveoli, determined by the connective tissue within the alveolar septa as well the surface tension properties of the air–liquid interface^40^. A schematic illustration of the how acinar conformation changes result in different exponents in *S* = *kV*^*n*^ is provided in Fig. 5.

### Conclusions

We developed a 4D synchrotron phase-contrast micro-CT technique allowing for the in vivo assessment of the expansion of lung acini with an isotropic 6 µm^3^ voxel size at 78 time points per breath, which to our knowledge is the highest achieved so far in dynamic imaging. We found that under the applied mechanical ventilation settings, regional lung strain within the lung peripheral airspaces took values of 0.09 ± 0.02 in anesthetized rats. Our data suggest that the conformational changes in lung airspaces are indicative of predominant alveolar expansion rather than ductal expansion or alveolar recruitment. We further analyzed the deformation in pulmonary blood vessels and found a large disparity depending on caliber. We believe this methodology will be instrumental in advancing our understanding of the dynamics of stress and strain within the lung parenchyma under mechanical ventilation and devising ventilation modes to preserve the lung parenchyma from stretch-induced injury.

## Methods

### Synchrotron radiation imaging

The experiments were performed at the biomedical beamline (ID-17) of the European Synchrotron Radiation Facility (ESRF, Grenoble, France). Briefly, X-rays produced by a multipole wiggler were monochromatized using a double bent Laue Si (111) monochromator^41^, selecting a photon energy of 40 keV. Monochromatic radiation impinged on the sample 146 m from the source and was detected by a fast Complementary metal–oxide–semiconductor (CMOS) PCO Edge 5.5 camera (PCO AG, Kelheim, Germany) coupled to a 250 μm thick Cerium-doped Lutetium Aluminum Garnet (LuAG:Ce) scintillator and optics yielding an isotropic pixel size of 6 μm^2^. The field of view of the camera was reduced to 15.4 × 1.2 mm^2^ to achieve a frame rate of ~ 500 fps, as a compromise between field of view and temporal resolution. The sample to detector distance was set to 4 m to utilize propagation-based phase contrast imaging^42^.

### Animal preparation

The care of animals and the experimental procedures were in accordance with the Directive 2010/63/EU of the European Parliament on the protection of animals used for scientific purposes, complied with the ARRIVE guidelines^43^ and were approved by the ESRF Ethical Committee on Animal Experimentation (Comité d'éthique en expérimentation animale; ETHAX #113) and the French Ministry of Higher Education and Research under the number: 2017071816174477. The study was performed on 3 female OFA rats (Ratus Norvegicus, Wt: 253.7 ± 7.2 g). Anesthesia was induced by intra-peritoneal injection of 1.5 to 2 ml kg^−1^ body weight of a solution containing Ketamine (40 mg ml^−1^) and Xylazine (20 mg ml^−1^). The animal was tracheostomized and a 14 G polyethylene catheter (Venflon; Becton Dickinson, Le Pont-de-Claix, France) was inserted and secured with a gas-tight seal. A 26 G polyethylene catheter (Neoflon; Becton Dickinson, Helsingborg, Sweden) was inserted in the jugular vein for fluid replacement (Lactated Ringer’s, B.Braun, France, 1–2 ml/kg/h). After surgery, the animal was immobilized in the vertical position in a custom-made plastic holder and placed on a remote-controlled rotation stage in the experimental hutch for imaging. Anesthesia was maintained during the duration of the experiment with 1% inhaled isoflurane (Isoflurane Belamont, Piramal Critical Care). The animals were mechanically ventilated with a tidal volume of 8 ml kg^−1^, FiO2 = 0.5; PEEP = 6 cmH_2_O at baseline. After verifying adequate depth of anesthesia (heart rate stability, inhibition of response to limb stimulation), muscle relaxation was induced by Atracurium injection (4 mg kg^−1^) to avoid motion and suppress spontaneous breathing. The electrocardiogram (ECG), was recorder by placing subcutaneous needle electrodes on the paws, and the neck, connected to a differential amplifier (Bio Amp, Adinstruments, Dunedin, New Zealand). ECG, airway pressure and air flow were continuously sampled at 10 kHz and recorded using a Powerlab 16/35 data acquisition device (DAQ, Adinstruments, Dunedin, New Zealand) (FigureS1).

### Image acquisition protocol

The image acquisition protocol was described in detail in a previous publication^44^. It was based on the synchronization between the heartbeat and the mechanical ventilation, which ensured a periodic lung parenchymal motion, as required by dynamic CT acquisitions. Briefly, the ECG signal was processed in real-time with a peak detector, which generated a square signal of predefined length and duty cycle when a R wave was detected, at the time instant *t*. The square signal was transmitted to the mechanical ventilator controller, to trigger an assisted breath. The R wave detection was disabled for the whole duration of the square signal. In the specific experiment discussed here, the square signal duration was $T=0.75 s$ and the heartbeat period was ${T}_{ECG}=0.39\pm 0.01$ s, implying ${n}_{ECG}= 2$ heartbeats per respiratory cycle. A full tomographic scan consisted of a set of 250,000 projections acquired with an integration time of 2 ms, over 180 degrees during a single sample half-rotation. A constant angular velocity, $\omega =0.34$° s^−1^ was set to include approximately 700 respiratory cycles per scan, over a total duration of 8.8 min.

### Image reconstruction

**Figure 6:**
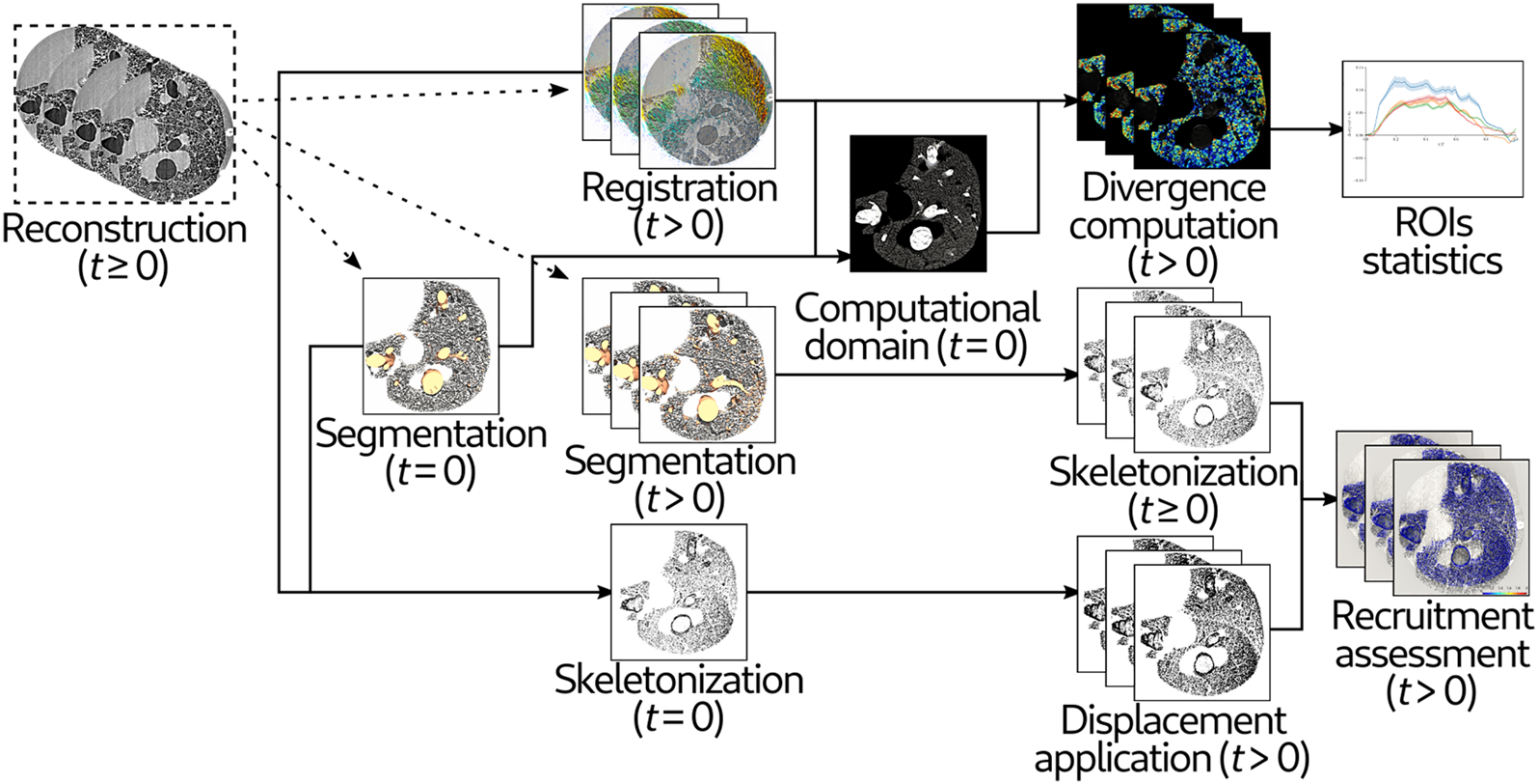
Image processing scheme. Sequential images were reconstructed at successive phases of breathing and heart contraction. Pairwise image registrations were performed between each time step and the immediately previous one. Deformation as a result of breathing or cardiac activity was computed and mapped. The presence of alveolar recruitment was investigated by comparing segmentations of the airspaces at each time step to the segmentation at time t = 0, properly realigned according to the results of image registration.

The image processing workflow is shown in Fig. 6. A retrospective gating methodology^44^ was used to reconstruct 4D CT images. In this approach each projection: $P\left( {\alpha ,{ }t^{*} } \right)$, is characterized by an angle, $\alpha = \omega \left( {t - t_{0} } \right)$, and a time: $t^{*} = t - t_{{j{ }n_{ECG} }}$, where $t_{{j{ }n_{ECG} }}$ is the time at which jth respiratory cycle was started:

1 $$t_{{j{ }n_{ECG} }} < t < t_{{\left( {j + 1} \right){ }n_{ECG} }}$$

The projections corresponding to the same time interval were sorted in 3D image stacks discretizing $t^{*}$ with a ${\Delta }t = 10$ ms time resolution. Tomographic reconstruction was performed with a Filtered Back Projection algorithm using PyHST2^45^ software, after application of Paganin’s single material phase retrieval algorithm^46^. Cupping artefact which frequently occurs in local tomography where the object is larger than the field of view, was dealt with using the STRAIGTHEN_SINOS option in PyHST2. Since the ECG signal is not perfectly periodic, the projections were not equally spaced. Hence, 78 3D-CTs were finally reconstructed.

### Image segmentation

The same segmentation process was used for blood vessels and air airspaces. Otsu's algorithm allowed for a first partitioning of air and non-air voxels^47^, based on the image histogram. To classify the former voxels as proximal, intermediate and terminal, an iterative process was performed. The axial regions of the proximal bronchi were identified by carrying out a stepwise 3D erosion process. Assuming the proximal bronchi are quasi cylindrical, these bronchi were identified by linear regression as regions where volume will decrease linearly with respect to the number of erosion steps, based on the root mean square error. The full volume of primary bronchi was then recovered by morphological dilatation. Intermediate structures were subsequently identified as structures requiring less erosion steps than the primary bronchi. All the selected voxels which were not classified as proximal or intermediate were classified as terminal structures, with a similar approach as the ones described previously in the literature^48,49^. Using this method, terminal airways as well as the proximal and intermediate blood vessels were identified. Occasionally, additional manual editing was performed as necessary by adding markers on the image and carrying out a watershed.

### Image registration

A non-rigid registration algorithm was used in order to deform the segmented airspace or vascular volumes. The registration algorithm applies a B-spline grid to represent the original segmented greyscale volume. It was initially proposed by Klein et al.^50^ and is published within the Python Image Registration Toolkit (PIRT)^51^. A Sum of Squared Differences (SSD) similarity metric was applied, as well as a Laplacian regularization term. The registration algorithm was applied in a pairwise fashion, between each time step and the immediately previous one, to compute the displacement field ${\varvec{u}}^{*} \left( {{\varvec{x}};\left( {n - 1} \right){\Delta }t,{ }n{\Delta }t} \right)$. This displacement field was then converted to a Lagrangian displacement field, representing the deformation between each image with the initial one at the start of the breath, through the following recursive process:

2 $${\varvec{r}}\left( {n{\Delta }t} \right) = {\varvec{x}} + {\varvec{u}}\left( {{\varvec{x}};0,n{\Delta }t} \right)$$

3 $${\varvec{u}}\left( {{\varvec{x}};0,n{\Delta }t} \right) = { }{\varvec{u}}\left( {{\varvec{x}};0,\left( {n - 1} \right){\Delta }t} \right) + {\varvec{u}}^{*} \left( {{\varvec{r}}\left( {n{\Delta }t} \right);\left( {n - 1} \right){\Delta }t,n{\Delta }t} \right)$$

where ***x*** is a point in the 3D space, ${\Delta }t$ is the time step between 2 images in the respiratory cycle, and *n* the number of images from the start of the breathing cycle. This two-step computation of the displacement field allowed to minimize registration errors. The error value was defined, as:

4 $$\varepsilon_{u} \left( {\varvec{x}} \right) = { }\frac{{{\varvec{u}}\left( {{\varvec{x}};0,T} \right) - {\varvec{u}}^{*} \left( {{\varvec{x}};0,T} \right)}}{{{\text{max}}\left( {{\varvec{u}}^{*} \left( {{\varvec{x}};0,T} \right)} \right)}}$$

which was used to assess the quality of the registration.

We investigated whether completely deflated airspaces were inflated upon inspiration, a phenomenon referred to as “recruitment”. To this end, we used skeletonization, a process that reduces binary objects to 1-pixel width representations, in order to extract the airspace topology. This was performed with the Skeletonize_3D toolkit (https://scikit-image.org)^52^. Skeletons were produced for the segmented airspace volume at each time instant ${ }s\left( {{\varvec{x}};{\Omega }\left( {n{\Delta }t} \right)} \right)$. The skeleton of the first time instant was deformed according the Lagrangian displacement field, $s\left( {{\varvec{r}}\left( {n{\Delta }t} \right);{\Omega }\left( 0 \right)} \right)$^53^. Each pair of skeletons, $s\left( {{\varvec{x}};{\Omega }\left( {n{\Delta }t} \right)} \right)$ and $s\left( {{\varvec{r}}\left( {n{\Delta }t} \right);{\Omega }\left( 0 \right)} \right)$ were compared by computing 2 distances, which were the distance between the skeleton and the segmented volume boundary:

5 $$d_{{\Omega }} = {\text{min}}\left\{ {s\left( {{\varvec{x}};{\Omega }\left( {n{\Delta }t} \right)} \right),{ }\partial {\Omega }\left( {n{\Delta }t} \right)} \right\}c$$

and the distance between the two skeletons:

6 $$d_{{\text{s}}} = {\text{min}}\left\{ {s\left( {{\varvec{x}};{\Omega }\left( {n{\Delta }t} \right)} \right),{ }s\left( {{\varvec{r}}\left( {n{\Delta }t} \right);{\Omega }\left( 0 \right)} \right)} \right\}$$

Recruited structures could then be eventually segmented by selecting initial seeds or voxels where $d_{{\text{s}}} > d_{{\Omega }}$. However, in this experiment, no recruited airspaces were detected.

### Computation of volume and surface change

The divergence theorem was applied to compute the volume change:

7 $${\Delta }v\left( {n{\Delta }t} \right) = \mathop \sum \limits_{{i \in {\Omega }}} \nabla \cdot {\varvec{u}}\left( {{\varvec{x}}_{i} ;0,n{\Delta }t} \right)$$

with ${\Omega }$ the segmented voxels at the first time point in the respiratory cycle, and ***x***_***i***_ the center of the *i*th voxel. The divergence theorem states that the divergence of the deformation field is equal to the voxel surface multiplied by the displacement distance of the corresponding voxel surface, which yields ***Δv***. Volume change is therefore computed by multiplying the original volume by the divergence of the deformation field, while strain is given directly by the divergence. To overcome the limitations of traditional Jacobian computations^54^, a high order Mean Least Squares (MLS) kernel-based convolution was applied^55^, which grants linear consistency:

8 $$\langle \nabla \cdot {\varvec{u}}\left( {{\varvec{x}}_{i} ;0,n{\Delta }t} \right) \rangle = { }\mathop \sum \limits_{{j \in {\Omega }}} {\varvec{u}}\left( {{\varvec{x}}_{j} ;0,n{\Delta }t} \right) \cdot \left( {L\left( {{\varvec{x}}_{i} } \right) \cdot \nabla W\left( {{\varvec{x}}_{j} - {\varvec{x}}_{i} } \right)} \right)$$

where W is the kernel convolution, and L is the MLS matrix which corrects the kernel compact support truncation:

9 $$L\left( {{\varvec{x}}_{i} } \right) = \left[ {\mathop \sum \limits_{{j \in {\Omega }}} \left( {{\varvec{x}}_{j} - {\varvec{x}}_{i} } \right) \otimes \nabla W\left( {{\varvec{x}}_{j} - {\varvec{x}}_{i} } \right)} \right]^{ - 1}$$

It should be noted that the convolution above considers only the segmented voxels. The computed maps of local strain at each time point within the respiratory cycle were then rendered using ParaView software^56^.

For the surface change computation, a similar expression to Eq. (7) was used, also based on the divergence theorem:

10 $${\Delta }s\left( {n{\Delta }t} \right) = \mathop \sum \limits_{{i \in {\Omega }}} \langle \nabla \cdot \left( \langle{h\left( {\varvec{x}}_{i} \right)\rangle {\varvec{u}}\left( {{\varvec{x}}_{i} ;0,n{\Delta }t} \right)} \right) \rangle$$

With *h* the mean Gaussian curvature^57^, which represents how the surface changes with respect to volume^57^.

## Supplementary Information

Supplementary Video 1.

Supplementary Video 2.

Supplementary Video 3.

Supplementary Information 1.

## Data Availability

Data and source code are available upon reasonable request to the corresponding author.
