# Supplementary material for: Lung tissue biomechanics imaged with synchrotron phase contrast microtomography in live rats
Source: Sci Rep. 2022 Mar 23;12:5056. doi: 10.1038/s41598-022-09052-9 (PMC8942151; doi:10.1038/s41598-022-09052-9)
Supplement: Supplementary file 4 — Supplementary Information 1. [file 41598_2022_9052_MOESM4_ESM.docx]

**Lung tissue biomechanics imaged with synchrotron phase contrast microtomography in live rats**

Jose-Luis Cercos-Pita^1*^, Luca Fardin^2*^, Hugo Leclerc^3^, Bertrand Maury^4^, Gaetano Perchiazzi^1^, Alberto Bravin^5^ & Sam Bayat^6,7^

^1^Hedenstierna Laboratory, Department of Surgical Sciences, Uppsala University, Uppsala, Sweden

^2^European Synchrotron Radiation Facility, Grenoble, France

^3^Laboratoire de Mathématiques d’Orsay, Université Paris-Saclay, Orsay, France

^4^Département de Mathématiques Appliquées, Ecole Normale Supérieure, Université PSL, Paris, France

^5^Milano Bicocca University, Physics Department, Milan, Italy

^6^Univ. Grenoble Alpes, Synchrotron Radiation for Biomedicine STROBE Inserm UA07, Grenoble, France

^7^Grenoble University Hospital Department of Pulmonology & Clinical Physiology, Grenoble France

*: These authors contributed equally to this work.


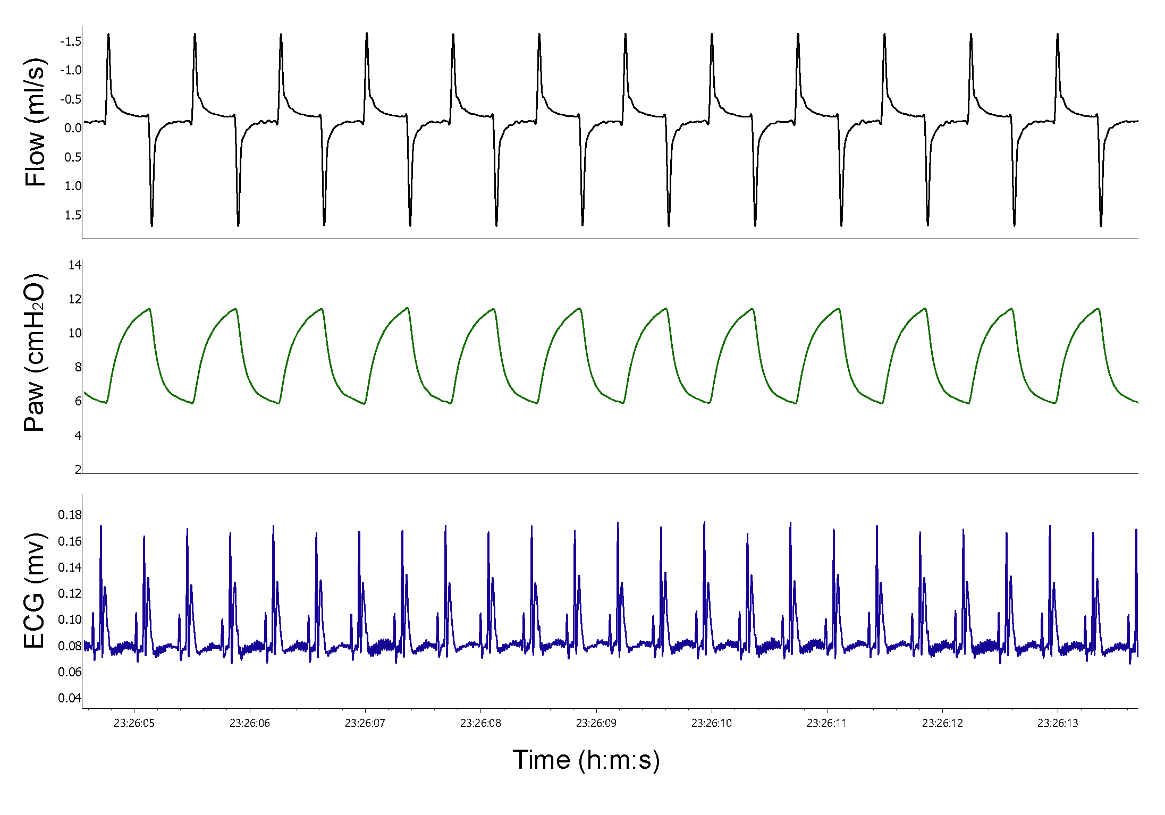


**Supplemental Figure S1.** Sample tracings of respiratory flow, airway pressure (Paw) and electrocardiogram (ECG) in a sample animal during imaging.

**Video animation 1.** Animated phase contrast CT images in a representative rat during a single breath: inspiration followed by expiration. Note the rib motion, and the large lung tissue deformations due to cardiac and vascular motion during two subsequent cardiac cycles occurring within the breath.

**Video animation 2.** Animated local strain maps of blood vessels in the same animal as video animation 1, during a single breath, computed based on elastic registration of successive images. Color bars indicate strain *(δV/V_t0_,* where *t0* is the start of the breath).

**Video animation 3.** Animated local strain maps of lung airspaces in the same animal as video animation 1, during a single breath, computed based on elastic registration of successive images. Color bars indicate strain *(δV/V_t0_,* where *t0* is the start of the breath).
